# Egg Food Challenges are Associated with More Gastrointestinal Reactions

**DOI:** 10.3390/children2030371

**Published:** 2015-08-17

**Authors:** Malika Gupta, Liron D. Grossmann, Jonathan M. Spergel, Antonella Cianferoni

**Affiliations:** 1 Department of Pediatrics, Division of Allergy and Immunology, The Children’s Hospital of Philadelphia, 3550 Market Street, Philadelphia, PA 19104-4399, USA; E-Mails: Guptam1@email.chop.edu (M.G.); spergel@email.chop.edu (J.M.S.); 2 Department of Pediatrics, Perelman School of Medicine at University of Pennsylvania, Philadelphia, PA 19104-4399, USA; 3 Tel-Aviv University, Tel Aviv 69978, Israel; E-Mail: lirongr1@mail.tau.ac.il

**Keywords:** egg allergy, epinephrine, gastrointestinal, skin prick testing, oral food challenge

## Abstract

Egg allergy is a common pediatric allergy, and is usually outgrown by elementary school age. There is, therefore, a need to perform an oral food challenge (OFC) to establish the presence of food allergy to egg. In this study, we conducted a retrospective review of 2304 OFCs at a pediatric center and analyzed the severity of reactions during egg OFCs and compared them with other foods. The gastrointestinal system (GI) has been reported as more affected in egg food challenge. This study confirmed that 11% of patients undergoing egg OFC had GI symptoms *vs.* 7% undergoing food challenges for other foods or compared to milk, peanut and tree nut, individually. However, the involvement of lower respiratory tract was less frequent with egg than observed in peanut and tree nut OFC and similar to observed rate in milk. In conclusion, our study confirmed that OFC to egg causes more GI symptoms and less respiratory symptoms compared to other foods, in particular peanuts and tree nuts. However, 27% of children who failed egg OFC had lower respiratory tract reactions and required the use of epinephrine, similarly to children undergoing milk challenge.

## 1. Introduction

Food allergy is increasing with a reported prevalence of 6%–10% in preschoolers in the developed world and 7% in the developing nations [1,2,3]. This along with the increased awareness leads to the performance of an allergy evaluation. Allergy evaluation can involve extensive, and often unwarranted testing for food allergy [4]. Consequently, many children are on exclusion diets, which are known to be associated with detrimental effects on growth and psychological development [5,6,7,8]. There is, therefore, a need to return foods back in the diets of children in a timely manner when appropriate.

Immunoglobulin E (IgE) mediated hen’s egg allergy is amongst the most common food allergies [9] with a prevalence between 0.5% to 2.5% in young children [1,2,3]. Previous data has consistently showed resolution of egg allergy in childhood with limited prevalence in adulthood [9,10,11], hence, the need to perform oral food challenges (OFCs) in children with a history of egg allergy. Indeed, there is conflicting data about the actual age of resolution. Different studies suggest different ages of development of tolerance in majority of affected pre-schoolers ranging from three years [12] to 16 years [2,10,11].

The diagnosis of food allergy is made based on a clinical history of reaction, physical exam for atopic signs and testing to detect the presence of specific IgE to a suspected food [13]. Testing for specific IgE may be performed indirectly by skin prick testing (SPT) to standardized reagents, or directly by concentrations of allergen specific serum IgE (sIgE) may be measured *in vitro* [13]. Testing only establishes sensitization, it does not always relate with clinical reactivity to the food [14]. The gold standard for food allergy diagnosis is a double blinded placebo controlled OFC and it is routine clinical practice to use open OFCs to confirm food allergy [14].

There is no treatment for food allergy including egg allergy as specific immunotherapy is under development. Once diagnosed, management involves dietary exclusion of the food unless the development of tolerance can be established. Several studies have showed the detrimental effect of on the height and weight of children [4,8,15,16]. The importance of OFCs for evaluation of food allergy is increasing, not only because of increased prevalence, but also due to increasing number of children on exclusion diets based solely on the detection of specific IgE [4,15]. Safety issues are an important consideration in deciding who to challenge and where the challenge should be done. It is important that OFCs be conducted in a location that is well equipped to deal with anaphylactic reactions by physicians and staff who are well trained in dealing with such reactions [14,17,18].

OFCs can be associated with systemic and life threatening reactions with some studies quoting rates as high as 28% [19]. Stratification of risks associated with OFCs is therefore needed to guide decisions regarding choosing the right locations for OFCs and, to counsel the families about expected risks associated with OFCs [20].

Although SPT [21], food specific sIgE [19,22,23] and specific IgE—to total IgE ratio [24] are very useful in predicting those who will fail or pass the OFC; they have not been shown to predict severity of reactions during OFCs when used in isolation [19]. Other studies have showed that a model using clinical history in conjunction with test results have better predictive ability than using either factor in isolation [14,25], however they are often complex and their applicability in the general practice is limited. Previous studies have reported peanut [14,20,25] and wheat [20] as independent risk factors for more severe reactions. The risk of severe reactions associated with egg OFC is not well described. Even though egg allergy is amongst the top three most prevalent food allergens along with milk, and peanut, it has been rarely described as a cause of near fatal or fatal anaphylaxis which overwhelmingly are caused by peanuts, tree nuts and milk [26,27,28,29].

Most egg allergy resolves during childhood [12]. As in the case of other foods, SPTs and serum specific IgE levels are often used as the first line testing to diagnose egg allergy. A recently published meta-analysis including 24 studies reported pooled sensitivities of 92% and 93% and specificities of 58% and 49% for SPTs and sIgE respectively for egg [30] for diagnosing egg allergy. In a Spanish study [31], 94 patients all of who were less than 18 months of age, with sensitization to egg via SPT to egg protein with/without serum sIgE to egg, were made to undergo OFCs to egg. None of these patients had previously ingested egg. Almost 29% of these children tolerated cooked and raw egg when challenged between 12 and 18 months of age [31]. Therefore, OFCs may be necessary to determine the clinical relevance of sensitization in the absence of positive history, especially because in some studies reactions during OFC to egg were mild and mainly involved the GI tract. For example in Clark’s longitudinal study with 181 egg challenges [32], there were 89 total positive reactions, of which none were treated with epinephrine. Although three of these challenges required the use of a nebulized bronchodilator, which one could argue could be treated with epinephrine, which is still a small number.

However, OFCs with egg can cause severe reactions. In a study by Benhamou *et al.* [33] that linked egg white specific IgE to the severity of reactions to raw or cooked egg during OFCs, 13 (57% of all positive reactions) of their 51 egg challenges resulted in severe reactions. Similarly, Rolinck *et al.* [34] found that 57% of the egg OFCs reactions involved the cardiovascular system. In a study by Vazquez-Ortiz *et al.* [35], 48 of 82 egg allergic children experienced anaphylaxis at double blinded placebo controlled OFCs.

In this study, we compared specifically the outcome of egg OFC compared to other common food allergy to determine its safety.

## 2. Methods: Study Population and Oral Challenges

We performed a retrospective analysis of children who underwent OFCs to wheat, soy, milk, egg, peanut, seeds, tree nuts, fish, shellfish and other foods at The Children’s Hospital of Philadelphia (CHOP) Pediatric Day Medicine unit from August 2004 to December 2014. OFCs were performed to determine if a patient has developed tolerance to previously allergic food or to determine if a SPT or specific IgE was clinical relevant (the subject was avoiding the food due to atopic dermatitis and positive sensitization but no clear history of true allergic reaction). None of the reported OFCs were performed with the intention to initiate an oral desensitization protocol [20]. All children undergoing OFC had a measurement of IgE levels via SPT in the six months preceding the OFC.

OFCs were performed by starting with a dose of 0.1 mL, followed by 0.5, 1, 2.5, 5, 10, 30, 60, 120, and 240 mL for liquid foods. For solid foods, the challenge doses administered were 125 mg, 250 mg, 500 mg, 1 gm, 2 gm, 4 gm, to a maximum of 8 gm, and *ad libitum* In the case of egg, the dosing is in the form of whole egg powder to a cumulative dose of 4 gm using the above dosing route followed by one whole scrambled egg. In select cases, a lower starting dose (20–60 mg) was chosen for highly sensitive children. Each dose was administered with an interval of 15 to 20 min until *ad libitum* doses were reached or the patient experienced a reaction within 2 h of the last dose. Challenges were stopped for gastrointestinal reactions, respiratory, cardiovascular or neurologic symptoms, non-contact cutaneous reactions or multi-system reactions. All providers followed the same standardized protocol as described. All children held antihistamine use for at least five days prior to the OFC.

Clinical data of the patients’ serum food allergen–specific IgE antibody levels (sIgE), SPT results, previous history of systemic reactions, history of asthma or eczema, and oral challenge outcomes were collected with IRB approval as previously described. All investigations were approved by The Children’s Hospital of Philadelphia Internal Review Board.

Of the 2304 challenges analyzed, 14 challenges were declared “indeterminate”, where the patient did not complete the dosing. This was in most cases due to patient preference issues (taste, anxiety, *etc.*). These patients were excluded from this analysis.

## 3. Classification of Reactions

OFC outcomes were broadly classified into three categories—positive (any reaction), negative (no reaction) and indeterminate (where the patient did not complete the OFC due to personal preference, not due to a reaction). Challenges with positive outcome were further sub-classified as cutaneous, respiratory, gastrointestinal, gastrointestinal with cutaneous, cardiovascular, multi-system reactions or anaphylaxis. Cutaneous reactions encompassed urticaria, erythematous flushing, cutaneous angioedema, or flaring of atopic dermatitis on non-contact areas. Respiratory reactions consisted of rhinitis, sneezing, voice change, throat tightness, dyspnea, cough, wheeze, shortness of breath, or tachypnea. Gastrointestinal reactions consisted of abdominal pain, emesis, or diarrhea. Cardiovascular reaction included hypotension and neurologic reactions included fainting. Multi-system reactions were those involving two or more systems [18].

We used the definition of anaphylaxis from the most recent NIH Food allergy guidelines which state that anaphylaxis is probable when several criteria are satisfied [13,36]. These criteria are as follows: 1) presence of skin signs or symptoms, together with respiratory involvement or signs of organic dysfunction or hypotension, or 2) involvement of at least 2 organs or systems after recent exposure to an allergen, or 3) signs of organ dysfunction or hypotension after exposure to a known allergen.

## 4. Skin Testing and Serum IgE

SPTs were performed at a prior clinic visit by the prick method using commercial extracts (Greer Laboratories, Lenoir, NC, USA) and bifurcated needles. Maximal wheal and flare diameter was measured at 15 min [36]. Saline was used as the negative control. A wheal of ≥3 mm than the negative control, when accompanied by a flare, was considered positive. Histamine dihydrochloride (10 mg/mL) acted as the positive control. Serum samples were analyzed for allergen-specific, sIgE antibody concentrations by using the Pharmacia CAP system FEIA (Pharmacia and Upjohn Diagnostics, Uppsala, Sweden). The lower limit of detection was 0.35 kU/L.

## 5. Statistical Methods

The relationships between the different types of OFC outcomes and other characteristics in both groups of children were analyzed by calculating the odds ratio and confidence intervals using a univariate or multivariate logistic regression or Chi square where indicated [37]. All tests were performed with STATA (version 11.0 for Windows; STATA Inc., College Station, TX, USA).

## 6. Results

A total of 2304 OFCs were performed at CHOP from 2005 to 2014 using the IgE-mediated food allergy protocol. The foods being challenged were diverse, with egg (26.1%), peanut (18.8%), milk (17.9%) and tree nuts (13%) being the most frequently challenged foods. A total of 601 challenges were conducted with egg via the IgE protocol in a pediatric patient population which was predominantly male (70.5%). Peanut, milk, and other tested antigen subgroups demonstrated a similar demographic spread by gender. Patients who underwent egg challenges were younger (mean ± SD = 5.1 ± 2.9 years) compared to patients who underwent challenges to other foods (6.7 ± 3.4 years). Patients undergoing egg OFCs also had a significant lower prevalence of seasonal allergic rhinitis and higher prevalence of eczema, and previous reactions to the challenged food compared to patients who underwent food challenge to foods other than egg. The prevalence of asthma was similar in the two studied groups (Table 1).

**Table 1:** Demographics of patient group challenged for egg compared with patient groups challenged for other foods.

	All Foods (Not Egg)	Egg
**Male, no. (%)**	1171/1703 (69)	424/601 (70)
**Atopic history, no. (%)**		
Asthma	1021/1703 (60)	341/601 (57)
Atopic Dermatitis	80/1703 (46)	315/601 (52)
Seasonal Allergic Rhinitis	719/1703 (42)	99/601 (16)
**Previous reaction**	936/1434 (65%)	374/542 (69%)^#^
**Age (years), mean ± SD**	6.7 ± 3.4	5.1 ± 2.9 *
**Positive OFCs; age (years), mean ± SD**	6.3 ± 3.1	5.1 ± 2.8 *

* *p* < 0.001 T Test, ^#^ Chi Square Test.

**Figure 1:**
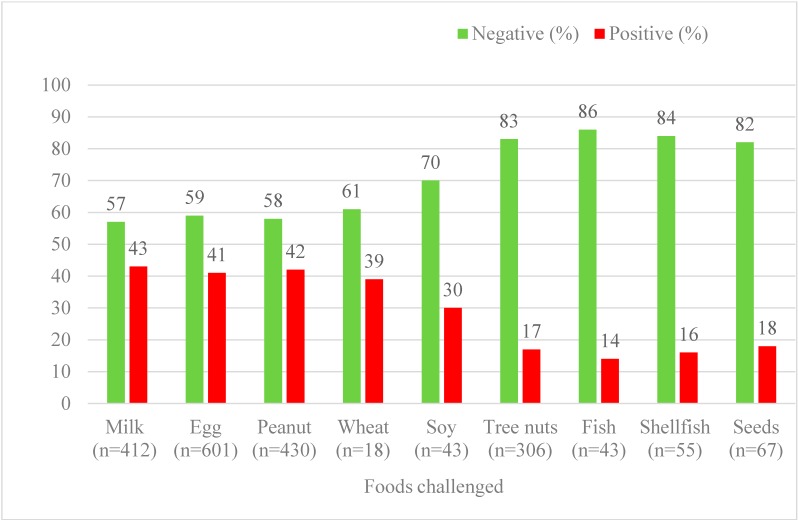
Rate of reaction on oral food challenges: Food challenges in the last 10 years at The Children’s Hospital of Philadelphia were compared. The percentage of oral food challenges with reaction (positive) and percentage with no reaction (negative) are shown. In addition, the number of challenges performed to each food group are listed.

The OFC outcomes of patients who were challenged with egg were compared with those patients who were challenged with other foods (Figure 1). The highest percentage of positive OFCs were with milk (178/412; 43.2%, *p* < 0.0001), egg (244/601; 40.6%, *p* < 0.0001), and peanut (180/430; 41.9%, *p* < 0.0001).

A significantly larger percentage of patients who underwent OFCs with egg had gastrointestinal symptoms compared to patients who underwent OFCs with other foods (11% *vs.* 7%, respectively, *p* < 0.001). There was no significant difference in age between those who had GI with or without cutaneous symptoms compared to those who had other (non-GI) symptoms (data not shown).

Skin prick testing results were available for 97.8% of the patients. As previously reported [14,20,21], those failing food challenges for egg had larger SPT wheal size with a mean of 6.5 mm (95% CI 6.0–6.9), but no statistical significant difference in the size of the wheal was observed between children requiring epinephrine (SPT wheal mean size of 6.9 mm ± 4.2) *vs.* those having milder reactions (SPT wheal mean size of 6.5 mm ± 3.1) (Table 2).

**Table 2:** Skin test of population with egg challenges based on outcome.

	Wheal (mm) Mean + Range	95% Confidence Interval
**Negative egg challenges**	4.4 + 2.8 *	4.1–4.7
**All positive egg challenges**	6.5 + 3.4 *	6.0–6.9
**Positive egg challenges, not requiring epinephrine**	6.5 + 3.1	6.0–7.1
**Positive egg challenges, requiring epinephrine**	6.9 + 4.2	5.8–7.9

* *p* < 0.0001 T Test.

Skin testing was performed using commercial whole egg extract. Egg OFCs were conducted with the use of raw egg powder followed by a scrambled egg.

Patients who failed challenges with egg had significantly less use of epinephrine than those undergoing food challenges with other foods (27% *vs.* 38%, *p* < 0.001). In comparing the most common four food allergens (egg, milk, peanut, tree nuts), isolated GI symptoms were more common in children with egg allergy compared to milk, peanuts and tree nuts, whereas lower respiratory symptoms were significantly less common than in children who failed tree nut and peanut OFC. No statistical significant difference was found in the prevalence of multi-system reactions (Table 3).

**Table 3:** Prevalence of gastrointestinal symptoms in positive OFCs: egg challenges compared with milk, soy, peanut or tree nuts.

	Egg, No. (%)	*Milk*	*Peanut*	*Tree Nuts*
**GI only**	37/244 (15%)	15/178 (8%) *	14/180 (8%) *	3/53 (7%) *
**GI + skin**	32/244 (13%)	23/178 (13%)	20/180 (11%)	6/53 (11%)
**Any GI**	69/244 (28)	42/178 (23%)	35/180 (20%) *	9/53 (17%)
**Lower respiratory**	65/244 (27%)	45/151 (30%)	60/139 (43%)	16/18 (88%) ^#^
**Multi-system**	144/244 (59%)	111/178 (62%)	116/180 (64%)	38/53 (71.7%)

* *p* < 0.05 Chi Square Test, ^#^ *p* < 0.001 Chi Square Test compared to frequency of reactions to egg.

The use of epinephrine was lower only compared to those children who failed OFC to peanuts and tree nuts but similar to the one for milk and in all failed OFC if there was a GI involvement. Therefore, the lower rate of epinephrine use could be explained by the lower rate of respiratory symptoms and not by the higher rate of GI reactions (Table 4).

**Table 4:** Epinephrine treatment in OFCs with positive (failed) outcome.

	Egg, No. (%)	Milk, No. (%)	Peanut, No. (%)	Tree nuts, No. (%)
**GI only**	1/37 (3%)	2/19 (10%)	1/15 (8%)	0/3 (0%)
**GI + skin**	7/32(22%)	4/23 (17%)	8/20 (40%)	3/6 (50%)
**Any GI**	8/69 (11%)	8/42 (19%)	9/35 (25%)	3/9 (33%)
**Any reaction**	66/244 (27%)	54/178 (30%)	76/180 (42.2%) ^#^	29/53 (55%) ^#^

^#^ *p* < 0.001 Chi Square Test.

## 7. Discussion

To our knowledge, this is the first study that compares specifically the outcome of egg OFCs to other foods. OFC data total of 2304 OFCs, of which 601 were to egg of the Division of Allergy and Immunology at CHOP from 2004 to 2014 were reviewed. Part of these data were analyzed previously and published [14,20]. Patients who underwent egg OFCs had a higher prevalence of atopic dermatitis (*p* < 0.001) than patients who underwent OFCs to other foods, consistent with the previously known association of egg allergy with atopic dermatitis [38,39]. Patients undergoing egg OFCs also had a lower prevalence of seasonal allergic rhinitis (*p* < 0.001), but a similar prevalence of asthma compared to patients undergoing OFCs with other foods. Additionally, patients undergoing egg OFCs were younger for all types of reactions. This is corroborated by the medical literature, which demonstrates that children with egg allergy, as compared to children with other allergies such as peanut or tree nut, outgrow their allergy earlier in life [12]. Additionally, the higher prevalence of atopic dermatitis in younger children compared with the higher prevalence of seasonal allergic rhinitis amongst children challenged at older ages is consistent with the natural history of atopy that evolves from food allergy and eczema into asthma and allergic rhinitis (“atopic march”) [40].

Of all conducted OFCs, the highest percentage of positive reactions were with milk followed by peanut and egg with the following failure rates for each food: milk 178 of 412 (43.2%), peanut 180 of 430 (41.9%), egg 244 of 601 (40.6%), wheat 7 of 18 (38.9%) and soy 13 of 43 (30.2%). Of these more common allergens, soy was associated with the lower risk of positive OFCs and milk with highest. This is consistent with our previously published data (2004) with no major change in recent years [41].

When we stratified our population on the basis of symptoms experienced during the challenge by system, we confirmed that a significantly larger percentage of patients who underwent egg OFCs had GI symptoms compared to patients who underwent OFCs with other foods. There was no significant difference in age between those who had GI symptoms and those who had other (non-GI) symptoms, suggesting that the GI involvement may be due to a specific reaction to egg and not being influenced by age. Other studies have also shown gastrointestinal symptoms to be a frequent complaint in egg OFCs [41]. Benhamou *et al.* [33] showed that 86% of their positive reactions had gastrointestinal complaints. Additionally in Clark *et al.*’s study [32], gastrointestinal symptoms were the second most commonly reported symptoms (reported positive egg challenges).

We also showed that patients who failed challenges with egg had significantly less use of epinephrine (27%) than those undergoing food challenges with other foods (38%). Compared to milk, peanuts and tree nuts, egg OFC had higher levels of isolated GI symptoms and lower levels of lower respiratory symptoms. This could explain the lower use of epinephrine in egg challenges compared to peanuts and tree nuts (Table 3 and Table 4).

Among children who reacted to egg, we evaluated if skin testing results could predict a more severe reaction. In our database, SPT (performed using commercial whole egg extract) results were available for 97.8% of the patients. As previously reported [21], those failing food challenges for egg had larger skin prick test. There was no statistically significant difference in the size of the wheal between those requiring epinephrine and those having milder reactions. In addition, the need for epinephrine did not show statistical difference for age.

In conclusion, we saw that 27% of children who failed egg OFC had severe symptoms, such as lower respiratory tract involvement and epinephrine use. Even though this percentage was lower compared to some foods, such as tree nuts and peanuts, it was similar to milk OFC outcome, and therefore egg OFC should be performed with caution. Our study, again, shows the limited usefulness of SPT in predicting severity of reactions.

We acknowledge the limitation of our study that it is a retrospective one. We also acknowledge that our study may have some selection bias as patients are selected for OFCs by experienced allergists based on SPT results, clinical history, and occasionally by sIgE results with reasonable avoidance of children who may have been prone to more severe reactions. Other limitation of our study is that comparison data for serum IgE levels were not available for most of our patients. Future studies that use all available diagnostic techniques in conjunction are required to predict severity of reactions during egg OFCs.

## Supplementary Files

Supplementary File 1
