# Supplementary material for: Egg Food Challenges are Associated with More Gastrointestinal Reactions
Source: Children (Basel). 2015 Aug 17;2(3):371–81. doi: 10.3390/children2030371 (PMC4928765; doi:10.3390/children2030371)

TABLE 1: Demographics of patient group challenged for egg compared with patient groups challenged for other foods

	**All Foods (not egg)**	**Egg**
**Male, no. (%)**	1171/1703 (69)	424/601 (70)
**Atopic history, no. (%)**		
Asthma	1021/1703 (60)	341/601 (57)
Atopic Dermatitis	80/1703 (46)	315/601 (52)
Seasonal Allergic Rhinitis	719/1703 (42)	99/601 (16)
**Previous reaction**	936/1434 (65%)	*374/542 (69%)
**Age (years), mean ± SD**	6.7 ± 3.4	5.1 ± 2.9*
**Positive OFCs; age (years), mean ± SD**	6.3 ± 3.1	5.1 ± 2.8*

*p<0.001

TABLE 2: Skin test of population with egg challenges based on outcome

	**Wheal (mm) mean ± SD**	**95% Confidence Interval**
**Negative egg challenges**	4.4 ± 2.8*	4.1-4.7
**All positive egg challenges**	6.5 ± 3.4*	6.0-6.9
**Positive egg challenges, not requiring epinephrine**	6.5 ± 3.1	6.0-7.1
**Positive egg challenges, requiring epinephrine**	6.9 ± 4.2	5.8-7.9

*p<0.0001 T Test

Skin testing was performed using commercial whole egg extract. Egg OFCs were conducted with the use of raw egg powder followed by a scrambled egg.

TABLE 3: Prevalence of gastrointestinal symptoms in positive OFCs: egg challenges compared with milk, soy, peanut or tree nuts

	**Egg, no. (%)**	***Milk***	***Peanut***	***Tree nuts***
**GI only**	37/244 (15%)	15/178 (8%)*	14/180 (8%) *	3/53 (7%)*
**GI + skin**	32/244(13%)	23/178 (13%)	20/180 (11%)	6/53 (11%)
**Any GI**	69/244(28)	42/178 (23%)	35/180 (20%)*	9/53 (17%)
**Lower respiratory**	65/244 (27%)	45/151 (30%)	60/139 (43%	16/18 (88%)#
**Multi-system**	144/244 (59%)	111/178 (62%)	116/180 (64%)	38/53 (71.7)

*$p<0.05$ #$p<0.001$ compared to frequency of reactions to egg

TABLE 4: Epinephrine treatment in OFCs with positive (failed) outcome

	**Egg, no. (%)**	***Milk, no. (%)***	***Peanut, no. (%)***	***Tree nuts, no.(%)***
**GI only**	1/37 (3%)	2/19 (10%)	1/15 (8%)	0/3 (0%
**GI + skin**	7/32(22%)	4/23 (17%)	8/20 (40%)	3/6 (50%)
**Any GI**	8/69 (11%)	8/42 (19%)	9/35 (25%)	3/9 (33%)
**Any reaction**	66/244 (27%)	54/178 (30%)	76/180 (42.2%)$^{\#}$	29/53 (55%)$^{\#}$

#-p<0.001

Figure 1:

Rate of reaction on oral food challenges: Food challenges in the last 10 years at The Children's Hospital of Philadelphia were compared. The percentage of oral food challenges with reaction (positive) and percentage with no reaction (negative) are shown. In addition, the number of challenges performed to each food group are listed.

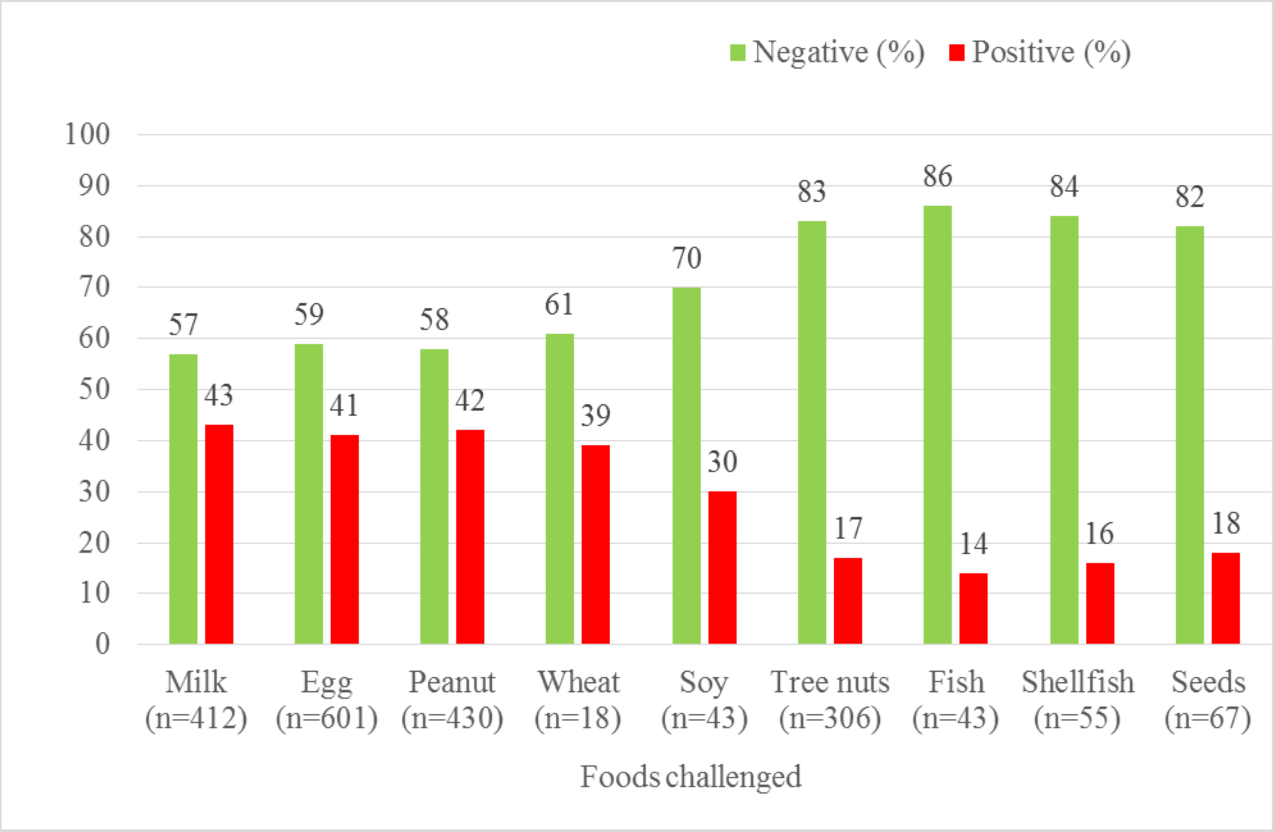

Supplement: Supplementary File 1 [file children-02-00371-s001.pdf]
